# Prevalence and type distribution of human papillomavirus (HPV) in Malaysian women with and without cervical cancer: an updated estimate

**DOI:** 10.1042/BSR20171268

**Published:** 2018-03-29

**Authors:** Shing Cheng Tan, Mohd Pazudin Ismail, Daniel Roza Duski, Nor Hayati Othman, Ravindran Ankathil

**Affiliations:** 1 UKM Medical Molecular Biology Institute, Universiti Kebangsaan Malaysia, 56000 Cheras, Kuala Lumpur, Malaysia; 2 Department of Obstetrics and Gynecology, School of Medical Sciences, Health Campus, Universiti Sains Malaysia, 16150 Kubang Kerian, Kelantan, Malaysia; 3 Department of Obstetrics and Gynecology, Hospital Sultan Ismail, 81100 Johor Bahru, Johor, Malaysia; 4 Department of Pathology, School of Medical Sciences, Health Campus, Universiti Sains Malaysia, 16150 Kubang Kerian, Kelantan, Malaysia; 5 Human Genome Centre, School of Medical Sciences, Health Campus, Universiti Sains Malaysia, 16150 Kubang Kerian, Kelantan, Malaysia

**Keywords:** Cervical cancer, Human papillomavirus, Prevalence, Type distribution

## Abstract

Information on the prevalence and type distribution of human papillomavirus (HPV) among Malaysian women is currently limited. The present study therefore aimed to provide an updated estimate on the prevalence and type distribution of HPV among Malaysian women with and without cervical cancer. Total DNA was isolated from the cervical cell specimens of 185 histopathologically confirmed cervical cancer patients and 209 cancer-free healthy females who were tested negative in a recent Pap test. Viral-specific DNA was subsequently amplified with biotinylated primers and hybridized to HPV type-specific probes via a proprietary “flow-through hybridization” process for determination of HPV genotype. It was demonstrated that 83.2% of the cervical cancer patients and none (0.0%) of the cancer-free females were positive for HPV infection. Among HPV-positive subjects, 14 different viral genotypes were observed, namely HPV16, 18, 31, 33, 35, 45, 52, 53, 58, 66/68, 73, 81, 82, and 84/26. A total of 91.6% of the HPV-positive subjects had single-type HPV infections and the remaining 8.4% were simultaneously infected by two HPV genotypes. The most common HPV infections found were HPV16 (35.7%), HPV18 (26.0%), HPV58 (9.1%), and HPV33 (7.1%) single-type infections, followed by HPV16 + HPV18 co-infections (5.2%). The study has successfully provided an updated estimate on the prevalence and type distribution of HPV among Malaysian women with and without cervical cancer. These findings could contribute valuable information for appraisal of the impact and cost-effectiveness of prophylactic HPV vaccines in the Malaysian population.

## Introduction

Cervical cancer is the seventh most common cancer worldwide and the fourth most common type of cancer among the female population [1]. Incidence data from GLOBOCAN database indicate that a total of 527,624 new cervical cancer cases were diagnosed in year 2012 alone, and approximately 84.3% of these occur in developing countries [1]. In Malaysia, the age-standardized rate of cervical cancer is 7.8 per 100,000 females, according to the most recent Malaysian National Cancer Registry Report [2].

Over the past decades, it has become established that human papillomavirus (HPV) is the central etiologic agent for cervical carcinogenesis [3]. The viral DNA has been shown to be present in virtually all cervical carcinoma specimens examined worldwide [4], and persistent infection of HPV has been definitively linked to the development of the cancer [3]. More than 200 types of HPV have been identified to date, which can be categorized into either cutaneous and mucosotropic type, with only the latter being relevant to the development of cervical carcinoma [5,6]. Mucosotropic HPV can further be classified into high and low risk groups, depending on their ability to immortalize human keratinocytes [6]. The high risk group includes HPV 16, 18, 31, 33, 35, 39, 45, 51, 52, 56, 58, 59, 68, 73, and 82 [7], and infection of any of these can result in cervical intraepithelial neoplasia, which can potentially progress into invasive cervical cancer.

HPV infection is preventable through vaccination. Several forms of HPV vaccines are currently available, which include a bivalent form (which targets on HPV16 and HPV18), a quadrivalent form (which targets on HPV6, HPV11, HPV16, and HPV18), and a nonavalent (9-valent) form (which targets on HPV6, HPV11, HPV16, HPV18, HPV31, HPV33, HPV45, HPV52, and HPV58) [8,9]. Understanding the pattern of HPV type distribution in a population is essential to help appraising the efficiency of the available HPV vaccines in protecting against the disease in the said population, since prevalence and type distribution of HPV are known to vary across different geographical regions [10,11]. For example, while the prevalence of HPV 16/18 was 67.2% among African women with cervical cancer, the percentage was 76.6% in the Oceania region [12]. Even within a continent, substantial difference could be observed. In Asia, for instance, the prevalence of HPV16/18 among cervical cancer cases was 65.0% in Eastern Asia, 70.4% in South-Eastern Asia, 80.3% in Southern Asia, and 72.4% in Western Asia [12].

The prevalence and type distribution of HPV in the Malaysian population are incompletely understood at the present. Several previous studies on these aspects in Malaysian population were either outdated [13], employed relatively small sample sizes [14,15], or only managed to identify a small number of HPV subtypes due to methodological limitations [13]. The present study attempted to overcome all these limitations by employing a relatively large sample size and utilizing a genotyping platform which can discern a large number of HPV subtypes. The objective of the present study was to provide an updated estimate of the prevalence and type distribution of HPV in Malaysian women with and without cervical cancer.

## Methods

### Ethical approval

The study received ethical approval from the Human Research Ethics Committee (HREC) of Universiti Sains Malaysia (reference numbers: USMKK/PPP/JEPeM [253.3.(7)] and USM/JEPeM/14100325) as well as the Medical Research and Ethics Committee (MREC) of Ministry of Health, Malaysia (reference numbers: KKM/NIHSEC/08/0804/P12-380 and KKM/NIHSEC/P15-1214). Written informed consent was obtained from all subjects prior to their enrolment into the study.

### Subject and sample collection

A total of 394 study subjects (comprising 185 cervical cancer cases and 209 cancer-free healthy females) were recruited from (i) Hospital Universiti Sains Malaysia, Kubang Kerian, Malaysia, (ii) Hospital Raja Perempuan Zainab II, Kota Bharu, Malaysia, and (iii) Hospital Sultan Ismail, Johor Bahru, Malaysia, between August 2012 and January 2016. The inclusion criterion for the cervical cancer cases was a clinical and histopathological confirmation of cancer of the uterine cervix. The histopathology analysis was performed on a biopsy specimen by a pathologist. On the other hand, healthy females were included if they were tested negative on a Pap test taken just before their enrolment into the study. The Pap test was performed by first collecting cervical cell specimen from the endocervical canal of the subjects with a sterile PathTezt^™^ Cervical Brush (Biocytech, Malaysia). While collecting the cervical cells, it was ensured that the shorter lateral bristles of the brush were in contact with the ectocervix. The cells obtained were suspended in cobas® PCR Cell Collection Media (Roche, Switzerland). A single layer of the cervical cells was then deposited on appropriate glass slides and the cells were examined through a computerized microscope with embedded camera. The residual cells suspended in the cell collection media were used for HPV typing if no abnormality was found in the Pap test. For patients with cervical cancer, cervical cells were collected by using the same method, but no Pap test was performed. Women with a previous history of cervical cancer or any other malignancy were excluded from the study.

### Isolation of DNA

Total DNA was isolated from cervical cell specimens by using QIAamp® DNA Mini Kit (QIAGEN, Germany). First, cervical cells were pelleted by centrifuging 1.5 ml of cell suspension at 13,200 rpm for 5 min. The process was repeated until a cell pellet of approximately 2.0 mm in diameter was obtained. The cervical cell pellet was then washed twice by centrifugation in 1.0 ml of phosphate-buffered saline at 13,200 rpm for 3 min. After the supernatant was discarded, the cell pellet was resuspended in 180 μl of Buffer ATL, and the remaining procedures of DNA isolation were then performed according to the manufacturer’s instructions. The concentration and purity of the DNA obtained were measured by using a spectrophotometer, and the quality of the DNA was checked with agarose gel electrophoresis. The total DNA isolated was subsequently diluted to a concentration of 20 ng/μl with an appropriate volume of nuclease-free water and stored at −20°C until used.

### Polymerase chain reaction with biotinylated primers

HPV-specific DNA present in the total DNA isolated was amplified with biotinylated PGMY primer set supplied in the PCR Kit of GenoFlow HPV Array Test Kit (DiagCor Bioscience, Hong Kong). All reaction volumes and conditions were based on the manufacturer’s instructions. A positive control and a negative control were prepared for each batch of sample run. Amplified HPV DNA was kept at 4°C until used.

### Probe hybridization and colorimetric signal development

After PCR amplification, the biotinylated HPV DNA amplicons were hybridized to membrane-coated HPV type-specific probes through a proprietary “flow-through hybridization” technology by utilizing the Hybridization Kit of GenoFlow HPV Array Test Kit (DiagCor Bioscience, Hong Kong). The “flow-through hybridization” and colorimetric signal development procedures were performed in a R2-M Flow-Through Hybridization System (DiagCor Bioscience, Hong Kong), based on the manufacturer’s instructions. HPV genotype was then determined by comparing the patterns of the colorimetric signal developed on the membrane with the reference guide supplied by the manufacturer. The assay was considered valid only if the amplification control (AC) and hybridization control (HC) signals were present, which respectively indicated a successful PCR reaction and probe hybridization.

## Results

### Characteristics of study subjects

A total of 185 cervical cancer cases and 209 cancer-free healthy females were recruited into the study. The demographic characteristics of the study subjects, including their age and ethnicity, are shown in Table 1. In addition, the FIGO stages and cancer histopathology of the cervical cancer cases are also presented in Table 1.

**Table 1:** Characteristics of study subjects

Characteristics	Cervical cancer cases (*N*=185)	Cancer-free females (*N*=209)
**Age (years)**		
Range	28–77	28–70
Mean ± SD	49.9 ± 10.4	45.6 ± 9.2
Median	48	46
**Ethnicity**		
Malay	124 (67.0%)	163 (78.0%)
Chinese	47 (25.4%)	35 (16.7%)
Indian	7 (3.8%)	9 (4.3%)
Others*	7 (3.8%)	2 (1.0%)
**FIGO stage**		
0	3 (1.6%)	–
IA1	11 (5.9%)	–
IA2	2 (1.1%)	–
IB1	32 (17.3%)	–
IB2	33 (17.8%)	–
IIA	29 (15.7%)	–
IIB	40 (21.6%)	–
IIIA	5 (2.7%)	–
IIIB	18 (9.7%)	–
IVA	8 (4.3%)	–
IVB	4 (2.2%)	–
**Histopathology**		
Squamous cell carcinoma	125 (67.6%)	–
Adenocarcinoma	47 (25.4%)	–
Neuroendocrine carcinoma	7 (3.8%)	–
Adeno-squamous carcinoma	2 (1.1%)	–
Serous papillary carcinoma	1 (0.5%)	–
Glassy cell carcinoma	1 (0.5%)	–
Mixed Müllerian tumor	1 (0.5%)	–
Mixed histopathological types^†^	1 (0.5%)	–

* Consisted of Cambodian (*N*=1), Eurasian (*N*=1), Thai people (*N*=3), Iban people (*N*=1), and Indonesian (*N*=1).

† Consisted of squamous cell carcinoma, adenocarcinoma, and small cell carcinoma histopathologies.

### Prevalence and type distribution of human papillomavirus in cervical cancer cases and cancer-free healthy females

The prevalence of HPV infection among cervical cancer patients and cancer-free healthy females is shown in Table 2. Among the cervical cancer cases, 154 (83.2%) were tested positive for HPV infection, while the remaining 31 (16.8%) were HPV-negative. On the other hand, all 209 (100.0%) cancer-free healthy females showed no detectable level of HPV infection. The data were also stratified by age group and cancer histopathology, and the results are shown in Table 2.

**Table 2:** Prevalence of human papillomavirus among the study subjects

Status	Prevalence	
	HPV positive	HPV negative
**Overall**		
Cervical cancer cases (*N*=185)	154 (83.2%)	31 (16.8%)
Cancer-free females (*N*=209)	0 (0.0%)	209 (100.0%)
**Cervical cancer cases only**		
*Stratified by age group*		
20–39 years (*N*=25)	22 (88.0%)	3 (12.0%)
40–59 years (*N*=123)	103 (83.7%)	20 (16.3%)
60–79 years (*N*=37)	29 (78.4%)	8 (21.6%)
*Stratified by cancer histopathology*		
Squamous cell carcinoma (*N*=125)	100 (80.0%)	25 (20.0%)
Adenocarcinoma (*N*=47)	43 (91.5%)	4 (8.5%)
Others (*N*=13)	11 (84.6%)	2 (15.4%)

The distribution of HPV subtypes among the infected subjects (in this case, the cervical cancer cases) is shown in Table 3. Of the 154 infected subjects, 141 (91.6%) had single-type HPV infections and the remaining 13 (8.4%) had simultaneous infections by two HPV subtypes. HPV16 and HPV18 were the most common HPV subtypes identified. Among subjects with single-type HPV infection, 55 (35.7%) and 40 (26.0%) had HPV16 and HPV18 infections, respectively. On the other hand, the most common multiple-type infection was HPV16 + HPV18 infections, accounting for 8 (5.2%) of all infected cases. With the exception of HPV84/26, all HPV subtypes identified were of “high risk” types. The distribution of HPV subtypes was also stratified by age group and subsequently by cancer histopathology, and the results are shown in Table 4.

**Table 3:** Distribution of HPV subtypes among infected subjects

HPV type	Classification*	HPV-positive subjects (*N*=154)	Percentage^†^
**Single-type infection**			
HPV16	HR	55	35.7
HPV18	HR	40	26.0
HPV31	HR	3	1.9
HPV33	HR	11	7.1
HPV35	HR	1	0.6
HPV45	HR	3	1.9
HPV52	HR	2	1.3
HPV53	HR	2	1.3
HPV58	HR	14	9.1
HPV66/68^‡^	HR	4	2.6
HPV73	HR	1	0.6
HPV81	HR	4	2.6
HPV82	HR	1	0.6
**Multiple-type infection**			
HPV16 + HPV18	HR + HR	8	5.2
HPV16 + HPV81	HR + HR	1	0.6
HPV16 + HPV84/26^‡^	HR + LR	1	0.6
HPV18 + HPV52	HR + HR	1	0.6
HPV18 + HPV58	HR + HR	1	0.6
HPV31 + HPV45	HR + HR	1	0.6

* Abbreviations: HR (high-risk HPV type); LR (low-risk HPV type).

† Total percentage is not equal to 100.0% due to rounding.

‡ The HPV genotyping kit used could not distinguish between HPV66 and HPV68, and between HPV84 and HPV26.

**Table 4:** Distribution of HPV subtypes in cervical cancer tissues of different histopathology based on age group

HPV types	Percentage of HPV infection (%)*								
	20–39 years (*N*=23)			40–59 years (*N*=103)			60–79 years (*N*=29)		
	SCC^†^ (*N*=14)	ADC^†^ (*N*=8)	Other (*N*=1)	SCC^†^ (*N*=67)	ADC^†^ (*N*=28)	Other (*N*=8)	SCC^†^ (*N*=20)	ADC^†^ (*N*=7)	Other (*N*=2)
**HPV16**	42.9	37.5	–	40.3	42.9	12.5	25.0	28.6	–
**HPV18**	21.4	50.0	100.0	20.9	25.0	50.0	25.0	14.3	50.0
**HPV31**	–	–	–	1.5	7.1	–	–	–	–
**HPV33**	14.3	–	–	11.9	3.6	–	–	–	–
**HPV35**	–	–	–	1.5	–	–	–	–	–
**HPV45**	–-	–	–	1.5	3.6	12.5	–	–	–
**HPV52**	–-	–	–	–	–	–	5.0	14.3	–
**HPV53**	–	–	–	–	–	–	10.0	–	–
**HPV58**	7.1	12.5	–	10.4	3.6	25.0	5.0	14.3	–
**HPV66/68****^‡^**	7.1	–	–	3.0	–	–	5.0	–	–
**HPV73**	–	–	–	–	–	–	5.0	–	–
**HPV81**	–	–	–	–	7.1	–	5.0	14.3	–
**HPV82**	–	–	–	–	–	–	–	–	50.0
**HPV16 + 18**	–	–	–	6.0	3.6	–	10.0	14.3	–
**HPV16 + 81**	–	–	–	1.5	–	–	–	–	–
**HPV16 + 84/26****^‡^**	7.1	–	–	–	–	–	–	–	–
**HPV18 + 52**	–	–	–	–	3.6	–	–	–	–
**HPV18 + 58**	–	–	–	–	–	–	5.0	–	–
**HPV31 + 45**	–	–	–	1.5	–	–	–	–	–

* Total percentage may not be equal to 100.0% due to rounding.

† Abbreviations: ADC, adenocarcinoma; SCC, squamous cell carcinoma.

‡ The HPV genotyping kit used could not distinguish between HPV66 and HPV68, and between HPV84 and HPV26.

## Discussion

In the present study, the prevalence of HPV in cervical cell specimens of cervical cancer patients was determined to be 83.2%. Since HPV has been established as an essential cause of cervical cancer [3], a prevalence of 100% is definitely expected in all cervical cancer specimens [12]. A prevalence of lower than 100% (as observed in this study) is most likely attributable to the limitations of the study methodology [12]. In particular, several possible reasons for the underestimation of HPV prevalence in the present study include (i) the failure to amplify HPV DNA of low copy numbers due to the intrinsic drawbacks of the PCR technique [16], (ii) the disruption of PCR primer target sequences due to the integration of HPV DNA into the host genome [17], (iii) the appearance of HPV DNA in the form of episome, which could not be extracted through the protocol used in the present study [18], (iv) the cells used for the HPV typing analysis which may have arisen from adjacent noncancerous tissues, rather than the cancerous portion of the cervix [19], and (v) suboptimal handling and storage of the specimens, especially during their transportation from the collaborating hospitals to the laboratory. In fact, underestimation of HPV prevalence seemed to be a common phenomenon in many studies—even the landmark paper by Walboomers et al. [4] reported a HPV prevalence of only 99.7% in cervical carcinomas, and several other prominent reports documented HPV prevalence rates of 96.0% [20], 92.9% [21], 89.9% [22], 87.2% [23], 87.0% [24], 85.0% [25], and 84.9% [26] respectively. Additionally, all previous studies in the Malaysian population also reported a less-than-100-percent prevalence of HPV in cervical cancer specimens, which ranged from 69.0% to 96.0% [13,15,27–29]. Besides, a study involving subjects from Southern Malaysia (Johor) and Singapore showed that the prevalence of HPV in cervical intraepithelial neoplasia (i.e. a precursor to cervical cancer) was 81.8% [30]. These observations suggested a reasonable agreement between the results obtained in the present work and those reported in previous studies.

On the other hand, among cancer-free healthy females, no HPV infection was detected in the present study. This observation was contrary to other reports in the Malaysian population. Chong et al*.* [31] reported a HPV prevalence of 46.7% among women without cytopathological sign of cervical neoplasia in Southern Selangor, while Othman and Othman [32] demonstrated a HPV prevalence of 3.1% among women with normal cytology in northeastern region of Peninsular Malaysia. Besides, the study by Tay and Tay [30] showed that 22.0% of cytologically normal women in Singapore and Johor had HPV infections. It can be postulated from these observations that obvious intracountry geographical variability exists in the prevalence of HPV among women without cervical neoplasia. This postulation could explain why the observation in the present study was the closest to that of Othman and Othman [32]—since majority of samples analyzed in this present work were also derived from patients in northeastern region of Peninsular Malaysia. In addition, careful selection of cancer-free females (i.e. based on the results of the most recent Pap test) represented another reason for the absence of HPV observed in the present study.

It was also demonstrated in the present work that majority (91.6%) of HPV infections were caused by a single type of HPV. This observation was in agreement with the findings reported by Quek et al*.* [28], which showed that 88.7% of HPV infections were single-type infections. Nevertheless, Hamzi Abdul Raub et al*.* [29] and Sharifa Ezat et al*.* [15] reported the contrary. The former showed that single-type infections accounted for only 39.8% of all HPV infections, while the latter found that 41.9% of the infections were caused by single-type HPVs. However, it is interesting to note that these two studies determined the HPV types utilizing a same commercial kit (HPV High Risk Typing Real-TM kit, SACACE, Italy), whose PCR primer sequences were not disclosed by the manufacturer. On the other hand, the PCR primer sequences used in the present study (the PGMY HPV primers) and that of Quek et al*.* [28] (the SPF10 HPV primers) were the ones which have been thoroughly validated and firmly established.

In the present study, it was also shown that single-type infection of HPV16 was the most common type of HPV infection detected, which accounted for 35.7% of all the infections. This was followed by single-type infection of HPV18 (26.0%), single-type infection of HPV58 (9.1%), single-type infection of HPV33 (7.1%), and multiple-type coinfection of HPV16+18 (5.2%). This observation was slightly different from the prevalence of HPV observed globally [12] as well as that reported in a few other previous studies in the Malaysian population [13,15,28,29,31,32] (Table 5). One possible reason for this discrepancy across the different studies could be, as discussed above, geographical variability in HPV type distribution. In addition, the study by Cheah et al*.* [27] suggested that the type distribution of HPV in a particular population may change over time, and the present study represented the latest estimation of HPV type distribution in the Malaysian population. Despite this, all the above reports showed that HPV16, HPV18, HPV33, and HPV58 were among the most common types of HPV observed across all study specimens (Table 5), which agreed with the findings of the present work. In addition to the above HPV types, the presence of HPV31, HPV35, HPV45, HPV52, HPV53, HPV66/68, HPV73, HPV81, HPV82, and HPV84/26 was also observed in the present work. Many of these HPV types corresponded to those targeted by the latest nonavalent (9-valent) HPV vaccine, which suggests that introduction and implementation of this new form of vaccine in Malaysia may greatly reduce the risk of cervical cancer among Malaysian women.

**Table 5:** The most common HPV types reported in previous Malaysian studies and in the present study

HPV genotype	Frequency (%)						
	Yadav et al. [13]*^,^^§^	Chong et al. [31]	Sharifa Ezat et al. [15]*	Quek et al. [28]	Othman and Othman [32]	Hamzi Abdul Raub et al. [29]*	Present study
HPV6	–	–	1.3	–	9.5	–	–
HPV11	–	–	1.3	–	–	–	–
HPV11/31/33/59^‡^	–-	–	–	1.0	–	–	–
HPV16	73.9	85.7	73.8	38.1	57.1	68.2	35.7
HPV18	65.2	7.1	22.5	26.8	4.8	40.0	26.0
HPV31/33^‡^	16.9	–	–	–	–	–	–
HPV31	–	1.2	1.3	–	–	0.7	1.9
HPV33	–	3.6	30.0	–	4.8	10.4	7.1
HPV35	–	–	1.3	–	–	1.4	0.6
HPV39	–	–	16.3	–	–	7.5	–
HPV45	–	–	13.8	7.2	–	9.6	1.9
HPV51	–	–	–	–	–	2.5	–
HPV52	–	–	16.3	10.3	–	10.4	1.3
HPV53	–	–	–	–	–	–	1.3
HPV56	–	–	–	3.1	–	7.1	–
HPV58	–	1.2	3.8	4.1	19.0	10.7	9.1
HPV59	–	–	2.5	–	–	5.7	–
HPV61	–	–	–	–	4.8	–	–
HPV66/68^‡^	–	–	–	–	–	–	2.6
HPV68	–	–	–	2.1	–	–	–
HPV73	–	–	–	–	–	–	0.6
HPV81	–	–	–	–	–	–	2.6
HPV82	–	–	–	–	–	–	0.6
HPV87	–	1.2	–	–	–	–	–
HPV16 + 18	–	–	–	–	–	–	5.2
HPV16 + 81	–	–	–	–	–	–	0.6
HPV16 + 84/26^‡^	–	–	–	–	–	–	0.6
HPV18 + 52	–	–	–	–	–	–	0.6
HPV18 + 58	–	–	–	–	–	–	0.6
HPV31 + 45	–	–	–	–	–	–	0.6

* The total frequencies in Baloch et al. [10], Bruni et al. [12], and Cheah et al. [27] were more than 100% as multiple infections were counted more than once. ^†^The total frequency in de Sanjose et al. [26] was less than 100%; no specific reason was given in their report.

‡ The HPV genotyping used in the respective studies cannot distinguish between the different HPV genotypes, or did not report the frequencies of the individual HPV types.

§ Only a small number of HPV genotypes were identified due to methodological limitations in this previous work.

Interestingly, the present work was the first study demonstrating the presence of HPV53, HPV73, HPV81, HPV82, and HPV84/26 (the HPV genotyping kit used could not distinguish between HPV84 and HPV26) in cervical epithelium of cervical cancer patients in the Malaysian population. None of the previous studies has reported the involvement of these HPV genotypes among cervical cancer patients and cancer-free healthy females in Malaysia. However, the observation of the present study was not surprising, as infections by all these HPV types have been documented formerly in other populations worldwide [12]. It can thus be postulated that the absence of these HPV infections in previous works in the Malaysian population could be due to limitations of the HPV genotyping method used. In fact, most HPV genotyping kits available presently can only discern between a limited types of HPV infections [16]. On the other hand, the HPV genotyping kit used in the present work can not only identify a panel of 33 different HPV genotypes, but also detect the presence of other HPV genotypes outside the above-mentioned panel [33].

In addition to high-risk HPV types, the genotyping kit used in the present work could also identify the presence of low-risk HPV types. The only low-risk HPV type identified in the present study was HPV84/26 (the HPV genotyping kit used could not distinguish between HPV84 and HPV26). Nonetheless, the role of HPV84/26 in mediating cervical carcinogenesis is likely limited, as HPV84/26 was noticed in a very low frequency of cervical cancer specimens not only in the present study (*N*=1), but also in other populations worldwide [12]. Moreover, infection of HPV84/26 observed in the present work was not a single-type infection, but rather, a multiple-type infection in conjunction with HPV16.

Besides, the findings on HPV prevalence and type distribution in the present work were also stratified by age group and cancer histopathology. Similar to several other large-scale previous studies [25,34–40], the results obtained in the present work showed a slight discrepancy in the prevalence and type distribution of HPV in subjects of different age groups and cancer histopathologies. Sample size appeared to be a major factor that contributed to this discrepancy, as the prevalence and type distribution of HPV in strata containing a larger number of samples resembled more closely to those of the overall (unstratified) analysis. This suggests that a large sample size is necessary for obtaining a consistent finding that may reflect the actual prevalence and type distribution in the population under study. The sample size employed in the present work is one of the largest ever investigated in the Malaysian population.

In the age-stratified analysis, it was observed that the prevalence rates of HPV infection generally decreased with age. This was in agreement with a global study involving more than 346,000 women from 70 countries, which showed a clear peak in HPV prevalence among women at late adolescence or early adulthood across all geographical regions [39]. The high prevalence of HPV infection among younger females of this age group could be explained by the fact that HPV is a sexually transmitted infection and majority of women commenced sexual activity during late adolescence or early adulthood [41–43].

Besides, stratification by cancer histopathlogy revealed that HPV positivity was more prevalent in adenocarcinomas (91.5%), compared with squamous cell carcinomas (80.0%) and cervical cancers of other histopathological types (84.6%). This observation was in contrast with the findings of many other previous studies [25,44–46]. It was postulated that the low prevalence of HPV in cervical adenocarcinoma in these previous studies could be attributed to the difficulty in sampling cervical columnar epithelial cells that give rise to the adenocarcinoma, as these cells are located deeper in the endocervical canal and are thus less accessible [25]. Nonetheless, the use of broom-type cervical brush, as in the present study, has been shown to significantly increase the probability of collecting adequate specimens from both ectocervical and endocervical components compared with other collecting devices such as spatula and cotton swab [47–48], which provides an explanation for the high rate of HPV infection detected in the present work.

There were few strengths and novelties in the present work. First, the present study employed a relatively large sample size (compared with other previous studies in the same population) for analysis, which could increase the reliability of the study findings. Besides, the present study utilized a HPV genotyping method which can discern diverse subtypes of the virus. Due to these reasons, several additional HPV subtypes (HPV53, HPV73, HPV81, HPV82, and HPV84/26) were demonstrated to be present for the first time in the cervical epithelium of Malaysian women.

## Conclusions

In conclusion, the present study has successfully provided an updated estimation of the prevalence and type distribution of HPV in Malaysian females with and without cervical cancer. These findings could contribute valuable information for appraisal of the impact and cost-effectiveness of prophylactic HPV vaccines in the Malaysian population.

## Abbreviations

ADC: adenocarcinoma
HPV: human papillomavirus
SCC: squamous cell carcinoma
